# Genomic resources in plant breeding for sustainable agriculture

**DOI:** 10.1016/j.jplph.2020.153351

**Published:** 2021-02

**Authors:** Mahendar Thudi, Ramesh Palakurthi, James C. Schnable, Annapurna Chitikineni, Susanne Dreisigacker, Emma Mace, Rakesh K. Srivastava, C. Tara Satyavathi, Damaris Odeny, Vijay K. Tiwari, Hon-Ming Lam, Yan Bin Hong, Vikas K. Singh, Guowei Li, Yunbi Xu, Xiaoping Chen, Sanjay Kaila, Henry Nguyen, Sobhana Sivasankar, Scott A. Jackson, Timothy J. Close, Wan Shubo, Rajeev K. Varshney

**Affiliations:** a Center of Excellence in Genomics & Systems Biology, International Crops Research Institute for the Semi-Arid Tropics (ICRISAT), Hyderabad, India; b University of Southern Queensland, Toowoomba, Australia; c University of Nebraska-Lincoln, Lincoln, USA; d International Maize and Wheat Improvement Center (CYMMIT), Mexico DF, Mexico; e Agri-Science Queensland, Department of Agriculture & Fisheries (DAF), Warwick, Australia; f Indian Council of Agricultural Research (ICAR)- Indian Agricultural Research Institute (IARI), New Delhi, India; g International Crops Research Institute for the Semi-Arid Tropics (ICRISAT), Nairobi, Kenya; h University of Maryland, MD, USA; i Center for Soybean Research of the State Key Laboratory of Agrobiotechnology and School of Life Sciences, The Chinese University of Hong Kong, Shatin, Hong Kong Special Administrative Region; j Crops Research Institute, Guangdong Academy of Agricultural Sciences, Guangzhou, China; k South Asia Hub, International Rice Research Institute (IRRI), Hyderabad, India; l Shandong Academy of Agricultural Sciences, Jinan, China; m Chinese Academy of Agricultural Sciences, Beijing, China; n Department of Biotechnology, Ministry of Science and Technology, Government of India, India; o National Centre for Soybean Research, University of Missouri, Columbia, USA; p Joint FAO/IAEA Division of Nuclear Techniques in Food and Agriculture, Vienna, Austria; q Bayer Crop Science, GA, USA; r University of California, Riverside, CA, USA

**Keywords:** Genomics, Sequencing, Genotyping platforms, Sequence-based trait mapping, Genomics-assisted breeding, Genomic breeding, Genomic selection

## Abstract

Climate change during the last 40 years has had a serious impact on agriculture and threatens global food and nutritional security. From over half a million plant species, cereals and legumes are the most important for food and nutritional security. Although systematic plant breeding has a relatively short history, conventional breeding coupled with advances in technology and crop management strategies has increased crop yields by 56 % globally between 1965−85, referred to as the Green Revolution. Nevertheless, increased demand for food, feed, fiber, and fuel necessitates the need to break existing yield barriers in many crop plants. In the first decade of the 21st century we witnessed rapid discovery, transformative technological development and declining costs of genomics technologies. In the second decade, the field turned towards making sense of the vast amount of genomic information and subsequently moved towards accurately predicting gene-to-phenotype associations and tailoring plants for climate resilience and global food security. In this review we focus on genomic resources, genome and germplasm sequencing, sequencing-based trait mapping, and genomics-assisted breeding approaches aimed at developing biotic stress resistant, abiotic stress tolerant and high nutrition varieties in six major cereals (rice, maize, wheat, barley, sorghum and pearl millet), and six major legumes (soybean, groundnut, cowpea, common bean, chickpea and pigeonpea). We further provide a perspective and way forward to use genomic breeding approaches including marker-assisted selection, marker-assisted backcrossing, haplotype based breeding and genomic prediction approaches coupled with machine learning and artificial intelligence, to speed breeding approaches. The overall goal is to accelerate genetic gains and deliver climate resilient and high nutrition crop varieties for sustainable agriculture.

## 1 Introduction

Systematic plant breeding began ∼200 years ago. The first artificial hybrid developed by crossing *Dianthus caryophyllus* and *D. barbatus* and the rediscovery of Mendel laws early in the twentieth century provided a jump start to modern genetics and breeding (Lee et al., 2015a). Although conventional breeding has a short history, improved irrigation systems, mechanization, the introduction of genetically improved varieties, and the usage of fertilizers and pesticides in agriculture led to increased global crop yields of 56 % between 1965−85, known as the Green Revolution. Global climate extremes, such as drought, flooding, extreme temperatures, and intensifying incidence of pests and diseases, especially during the last 40 years, have had a serious impact on agriculture and threaten the livelihoods of agricultural producers and the food security of communities (IFPRI, 2019; Janssens et al., 2020). In 2019 alone, 144 million children under 5 years of age were estimated to be stunted and 340 million children suffered from micronutrient deficiencies (http://www.fao.org/worldfoodsituation/csdb/en/) and numbers are expected to be worse for 2020 (https://www.un.org/en/chronicle/article/forecast-2020-financial-meltdown-and-malnutrition#:∼:text=Globally%2C%2016%20million%20more%20children,to%20one%2Dfourth%20in%202020). Among the 17 Sustainable Development Goals (SDGs), adopted by all United Nations Member States, achieving “Zero Hunger”, the SDG2 requires more efficient, sustainable, climate-smart and nutrition-sensitive agriculture and food systems.

From more than a half million plant species on earth, cereals and legumes are the most important for food and nutritional security. Cereals provide the majority of calories consumed around the world, while legumes are a critical source of protein and complement amino acid deficiencies of cereal crops. The major cereals grown in the world are rice (*Oryza sativa*), maize (*Zea may*s), wheat (*Triticum aestivum*), barley (*Hordeum vulgare*), sorghum (*Sorghum biocolor*) and pearl millet (*Pennisetum glaucum syn. Cenchrus americanus*). Among legumes, soybean (*Glycine max*), groundnut (*Arachis hypogaea*), common bean (*Phaseolus vulgaris*), cowpea (*Vigna unguiculata*), chickpea (*Cicer arietinum*) and pigeonpea (*Cajanus cajan*) are the most consumed. While cereal crop productivity has increased during the last 50 years, the growth rate in the legume crop productivity has remained relatively slow. However, more recently, the yields of major cereals and that of soybean are nearing a plateau with very small rates of increase. As described in the study of of Ray et al. (2013), yields in maize, rice, wheat, and soybean are increasing at 1.6 %, 1.0 %, 0.9 %, and 1.3 %, respectively per year, far below what is needed to meet projected demands in 2050. For instance, the average increase in rice production in the 1980s was 3.1 % per year, which dropped to 1.4 % per year in the 1990s, and 0.8 % per year in the 2000s (Phillips, 2010). Furthermore, yields of some crops like rice and wheat have plateaued in some of the highest yielding and highest input agricultural systems (Grassini et al., 2013). Increased demand for food, feed, fiber, and fuel necessitates the breaking of the existing yield barriers in different crop plants. Therefore, a coordinated effort of new agronomic methods, advancements in breeding technologies, development of novel genetic diversity and the utilization of genomic resources to discover and integrate novel genes and alleles are required to cope with the challenges facing crop production to achieve the goals of sustainable agriculture.

In the first decade of the 21st century we witnessed rapid discovery, transformative technological development and declining costs in the area of genomics. In the second decade, the field turned towards making sense of the vast amount of genomic information and accurately predicting and tailoring climate resilience of crops for global food security. Advances in next generation sequencing (NGS) technologies enabled the decoding of many crop genomes facilitating the development of molecular markers for use in trait dissection, trait selection as well as trait improvement (Bohra et al., 2020). Additionally, precision phenotyping (Giovanni and Murray, 2018; Yang et al., 2020), artificial intelligence (Beans, 2020) and genome editing (see Mackelprang and Lemaux, 2020; Baumann, 2020) are now being used in crop improvement.

Here we focus on genomic resources such as genome assemblies and germplasm sequencing, and their use for sequence-based trait mapping and genomic breeding, as they relate to developing climate resilient varieties in six major cereals (rice, maize, wheat, barley, sorghum and pearl millet), and six major legumes (soybean, groundnut, cowpea, common bean, chickpea and pigeonpea).

## 2 Assembling reference genome and germplasm sequencing

Arabidopsis (125 Mb) and rice (466 Mb) with small genomes among dicots and monocots, respectively, were forerunners as models for plant genetics and genomics research. Their genome sequences were first announced in 2000 for Arabidopsis and 2005 for rice (The Arabidopsis Genome Initiative, 2000; Sasaki, 2005). The reference genomes of these species played a critical role in shaping out our understanding of important genes and biological functions in these plant species. However, various plant species have unique features and different kinds of genome organization, including different ploidy levels and widely varying repeat contents. Plant communities began to assemble reference genomes for their respective species, a trend that accelerated in parallel with massive reductions in sequencing costs, the onset of single-molecule long-range sequencing and physical mapping, and improvements in genome assembly algorithms and pipelines. As a result, the draft genomes of over 800 plant species have been generated, and with steadily increasing genome assembly quality (both reduced error and increased contiguity) (Manchanda et al., 2020; Michael and VanBuren, 2020).

Besides the genomes of cultivated plant species, *de novo* assembled genomes of several wild relatives have also become available. Furthermore, the increasing realization that a single reference genome cannot represent the diversity present within a species has led to the increasing adoption of the concept of pan-genomes. Initially proposed in prokaryotic systems, a pan-genome aims to capture the total diversity within a species including both core genes shared by all sequenced individuals and dispensable genes which are present in some individuals but absent from others (Tettelin et al., 2005). The discovery that more than 10 % of high confidence maize genes present in the initial reference genome were absent in the genomes of one or more other maize inbreds (Swanson-Wagner et al., 2010) sparked interest in the contribution of plant pan-genomes to phenotypic variation in crop species. This has led to increased interest in developing pan-genome datasets, resources, and analysis tools (Tao et al., 2019). A number of these pan-genomes incorporate not only within species genomic content variation but also variation between the crop species and wild progenitors within the same genus, a type of pangenome sometimes referred to as a super pan genome (Khan et al., 2020).

In the following sections, we summarize the efforts on germplasm sequencing in the earlier mentioned cereal and legume species.

### 2.1 Cereals

Rice was the first crop plant to have an advanced draft genome sequence (Goff et al., 2002; Sasaki, 2005; see Jackson, 2016), and this work has continued with genomes now available for ssp. japonica (Longdao5; Jiang et al., 2017), multiple indica cultivars including 93–11, Nipponbare, DJ 123, Minghui 63 and Zhenshan 97, IR64, Shuhui498 in addition to platinum standard reference genomes (Yu et al., 2002; Kawahara et al., 2013a,b; see Sanchez et al., 2013; Schatz et al., 2014; Zhang et al., 2016; Du et al., 2017; Zhou et al., 2020). Draft genomes for two Australian wild A genome taxa, viz., *O. rufipogon*‐like population, referred to as Taxon A, and *O. meridionalis*‐like population, referred to as Taxon B (Brozynska et al., 2017) have also been assembled. Recently Shi et al. (2020) assembled 736.66 Mb genome of an endangered wild upland rice species, *O. granulata*, which provided novel insights into rice genome evolution, enhancing our efforts to search for new genes for future rice breeding programs and also facilitating the conservation of germplasm. Furthermore, sequencing of two wild rice lines (*O. rufipogon*, Huaye 1 and Huaye 2) identified NBS-LRR genes associated with disease resistance (Liu et al., 2017).

During the last decade, sequencing of germplasm lines such as a minicore collection (Kim et al., 2016), over 1000 indica accessions (Lv et al., 2020) and three basmati rice varieties (Kishor et al., 2020) provided greater insights into genome diversity, genetic diversity, structural variations, genes or novel genetic factors that potentially regulate important phenotypes different patterns of heterosis. Further, sequencing of genomes of 40 cultivated and 10 wild progenitors (*Oryza rufipogon* and *O. nivara*) of rice enabled identification of candidate regions selected during domestication, evident from thousands of genes with significantly lower diversity in cultivated but not wild rice (Xu et al., 2012). In addition, there has been sequencing of mutant lines (Li et al., 2019a) and biparental populations (Huang et al., 2009; Yang et al., 2017a), enabling identification of a mutation in *OsSh1* causing non-shattering in a rice (Li et al., 2020a) and fine mapping of QTLs (Kang et al., 2019). Based on whole genome sequencing of 3010 Asian rice germplasm accessions Wang and colleagues (2018a) reported 29 million SNPs, 2.4 million small indels, and ∼90,000 structural variations that contribute to within- and between-population variation. Further, a Rice Pan-genome Browser (RPAN; http://cgm.sjtu.edu.cn/3kricedb/ or http://www.rmbreeding.cn/pan3k) was developed that provides multiple search and visualization functions of genomic sequences, gene annotations, PAV information of 3010 accessions and gene expression data of the rice pan-genome (Sun et al., 2017). Similarly, sequencing of a germplasm set from the National Agriculture and Food Research Organization has enhanced understanding of the basis for diversity and as well as association of several seed-related phenotypes with known genes (Tanaka et al., 2020). Among several databases that make the data accessible to rice community (Table 1), RFGB v2.0 a comprehensive database with five major modules (Phenotype, Haplotype, SNP and InDel, Germplasm and Restore Sequence) enables haplotype mining (Wang et al., 2020).

**Table 1:** List of some omic databases in select cereal and legume crops.

Crop	Database	Salient feature	URL
Rice	RFGB database	RFGB v2.0 contains five major modules, including Phenotype, Haplotype, SNP & InDel, Germplasm and Restore Sequence.	http://www.rmbreeding.cn/
	R-PAN	rice pan-genome browser for ∼3000 rice genomes	http://cgm.sjtu.edu.cn/3kricedb/ or http://www.rmbreeding.cn/pan3k.
	RiceVarMap	The database provides comprehensive information of 6 551 358 SNPs and 1 214 627 InDels identified from sequencing data of 1479 rice accessions	http://ricevarmap.ncpgr.cn/v2/
	RiTE	Rice TE database (RiTE-db) - a genus-wide collection of transposable elements and repeated sequences across 11 diploid species of the genus *Oryza* and the closely-related out-group *Leersia perrieri*	https://www.genome.arizona.edu/cgi-bin/rite/index.cgi
	Rice Annotation Project Database (RAP-DB)	RAP-DB offers a highly reliable gene annotation based on the latest and most accurate genome assembly to date	https://rapdb.dna.affrc.go.jp/
	OryGenesDB	Insertion mutants of rice genes are catalogued by Flanking Sequence Tag (FST) information that can be readily accessed by this database	https://orygenesdb.cirad.fr/
	RiceSRTFDB	A database of rice TFs containing comprehensive expression, *cis*-regulatory element and mutant information to facilitate gene function analysis	http://www.nipgr.ac.in/RiceSRTFDB.html
	The Rice Genome Knowledgebase (RGKbase)	An annotation database for rice comparative genomics and evolutionary biology	http://rgkbase.big.ac.cn/RGKbase/
	Rice GT Database	Several classes of functional genomic data, including mutant lines and gene expression data, can be displayed for each rice glycosyltransferase (GT) in the context of a phylogenetic tree, allowing for comparative analysis both within and between GT families	https://ricephylogenomics.ucdavis.edu/cellwalls/gt/
	Rice GH Database	The database contains information on 614 putative rice GHs (gene models)	https://ricephylogenomics.ucdavis.edu/cellwalls/gh/
	Rice Transporter Database	The database contains information on 1754 putative rice transporters (gene models)	https://ricephylogenomics.ucdavis.edu/transporter/
	Rice TF Database	The database contains information on 3119 putative rice TFs (gene models)	https://ricephylogenomics.ucdavis.edu/tf/
	Rice Proteome Database	Database contains 23 reference maps based on 2D-PAGE of proteins from various rice tissues and subcellular compartments	http://gene64.dna.affrc.go.jp/RPD/
	OryzaPG-DB	The database contains the proteome and phosphoproteome of the rice undifferentiated cultured cells, the corresponding cDNA, Transcript an d Genome sequences, the novel proteogenomics features and the updated gene models annotation.	http://oryzapg.iab.keio.ac.jp/
	Rice SNP-seek	A new SNP-calling pipeline followed by filtering that resulted in complete, base, filtered and core SNP datasets.	https://snp-seek.irri.org/
	RiceXPro	The data base is a repository of gene expression profiles derived from microarray analysis of tissues/organs encompassing the entire growth of the rice plant under natural field conditions, rice seedlings treated with various phytohormones, and specific cell types/tissues isolated by laser microdissection (LMD).	https://ricexpro.dna.affrc.go.jp/

Maize	MaizeDIG	MaizeDIG is preloaded with 2396 images that are available on genome browsers for 10 different maize reference genomes	https://maizedig.maizegdb.org/
	MaizeGDB	MaizeGDB hosts a wide range of data including recent support of new data types including genome metadata, RNA-seq, proteomics, synteny, and large-scale diversity	https://www.maizegdb.org/
	MODEM	MODEM designed to promote a better understanding of maize genetic architecture and deep functional annotation of the complex maize genome and to explore the genotype–phenotype relationships and regulation of maize kernel development at multiple scales	http://modem.hzau.edu.cn/
	proFITS of Maize	Identify and classify protein kinases/phosphatases, TFs and ubiquitin-proteasome-system related genes in the B73 maize genome	http://bioinfo.cau.edu.cn/ProFITS/
	TIGR Maize Database	It has assembled and annotated the AZMs and used available sequenced markers to anchor AZMs to maize chromosomes.	http://maize.jcvi.org/
	Maize TE database	Stores information about transposable elements (TEs)	http://maizetedb.org/
	ZmGDB	A genomics database encompassing sequence data for green plants	http://www.plantgdb.org/ZmGDB/
	ZEAMAP	Functional annotations and comparative genomics of maize and teosinte genomes	http://www.zeamap.com/
	MaizeSNPDB	A comprehensive database for efficient retrieve and analysis of SNPs among 1210 maize lines	http://150.109.59.144:3838/MaizeSNPDB/
	PPIM	A protein-protein interaction database for Maize	http://comp-sysbio.org/ppim/

Wheat	CerealsDB	Genotyping information for over 6000 wheat accessions and describe new webtools for exploring and visualizing the data and also describe a new database of quantitative trait loci that links phenotypic traits to CerealsDB SNP markers and allelic scores for each of those markers.	https://www.cerealsdb.uk.net/cerealgenomics/CerealsDB/indexNEW.php
	PmiRExAt	A new online database resource that caters plant miRNA expression atlas	http://pmirexat.nabi.res.in/
	expVIP	Wheat transcriptome resources for expression analysis	http://www.wheat-expression.com/
	WheatExp	Homoeologue-specific database of gene expression profiles for polyploid wheat.	https://wheat.pw.usda.gov/WheatExp/
	WheatGenome	GBrowse2-based wheat genome viewer with BLAST search portal, TAGdb for searching wheat second-generation genome sequence data, wheat autoSNPdb, links to wheat genetic maps using CMap and CMap3D, and a wheat genome Wiki to allow interaction between diverse wheat genome sequencing activities	http://wheatgenome.info
	wDBTF	Collates 3820 wheat sTFs sequences	http://wwwappli.nantes.inra.fr:8180/wDBFT/
	MASWheat	Marker-assisted selection database for wheat	https://maswheat.ucdavis.edu/
	WISP	The Wheat Improvement Strategic Programme Consortium	http://www.wheatisp.org/
	OpenWildWheat	Sequencing resources of *Ae. tauschii* accessions	http://www.openwildwheat.org
	Wheat Atlas	Atlas of wheat germplasm and production statistics	http://wheatatlas.org
	WheatIS	An International Wheat Information System, to support the wheat research community	http://www.wheatis.org/
	Graingenes	Datasets useful to researchers working on wheat, barley, rye and oat	https://wheat.pw.usda.gov

Barley	barleyGenes	Provides access to the predicted genes from an assembly of whole-genome-shotgun sequence from barley	https://ics.hutton.ac.uk/barleyGenes/
	BARLEX	BARLEX was constructed to be the central repository and hub of genomic sequences of barley sequencing efforts.	https://apex.ipk-gatersleben.de/apex/f?p=284:10::::::
	bex-db	Bioinformatics workbench for comprehensive analysis of barley-expressed genes	https://barleyflc.dna.affrc.go.jp/bexdb/
	BRIDGE	A visual analytics web tool for Barley Genebank Genomics	https://bridge.ipk-gatersleben.de.

Sorghum	SorghumFDB	It constructed a dynamic network of multidimensional biological relationships, comprised of the co-expression data, protein–protein interactions and miRNA-target pairs	http://structuralbiology.cau.edu.cn/sorghum/index.html
	SorGSD	SorGSD has a web-based query interface to search or browse SNPs from individual accessions, or to compare SNPs among several lines.	http://sorgsd.big.ac.cn/
	Sorghum QTL Atlas	Integrated over 6000 QTLs previously described in sorghum for 220 traits and predicts syntenic locations in maize and rice	https://aussorgm.org.au/sorghum-qtl-atlas/
	SbGDB	Help annotate the *Sorghum bicolor* genome using yrGATE gene structure annotation tool	http://www.plantgdb.org/SbGDB/

Pearl Millet	PMDTDb	It catalogues the differentially expressed genes in response to drought along with TFs, gene regulatory network (GRN) having hub genes and genic region putative marker discovery (SSRs, SNP and Indels)	http://webtom.cabgrid.res.in/pmdtdb/

Soybean	SoyBase	SoyBase includes an extensive RNA-Seq gene atlas and innovative tools for identifying fast neutron-induced mutants	https://soybase.org/
	SoyGD	Tetraploid, octoploid, diploid and homologous regions are shown clearly in relation to an integrated genetic and physical map.	http://soybeangenome.siu.edu/
Groundnut	PeanutMap	An online genome database for comparative molecular maps of peanut	http://peanutgenetics.tamu.edu/cmap
	PeanutBase	Genetic and genomic data to enable more rapid crop improvement in peanut	https://peanutbase.org/home

Cowpea	CGKB	The CGKB consists of three knowledge bases: GSS annotation and comparative genomics knowledge base, GSS enzyme and metabolic pathway knowledge base, and GSS SSRs knowledge base for molecular marker discovery.	http://cowpeagenomics.med.virginia.edu/CGKB/
	EDITS	EDITS-Cowpea database is an open-access database that contains information relating to characteristics of cowpea	https://www.jircas.go.jp/en/database/edits-cowpea/introduction

Common bean	PhaseolusGenes	PhaseolusGenes is a web resource for identifying and exploring markers, QTL, and SSRs	http://phaseolusgenes.bioinformatics.ucdavis.edu/
	PvGEA	This atlas presents the gene expression patterns of 24 unique samples collected from seven distinct tissues of *Phaseolus vulgaris*cv. negro jamapa; roots, nodules, leaves, stems, flowers, seeds, and pods.	http://plantgrn.noble.org/PvGEA/

Chickpea	CicerTransDB	Provide a centralized putatively complete list of TFs in a food legume, chickpea and also genome-wide domain study and manual classification of TF families	http://www.cicertransdb.esy.es/
	Chickpea ISM-ILP Marker Database	This marker database contains genome-wide 119,169 and 110,491 ISMs from 23,129 desi and 20,386 kabuli protein-coding genes. It also catalogues 7454 in silico InDel, (1–45-bp)-based ILP markers from 3283 genes	http://webapp.cabgrid.res.in/chickpea/
	Integrated Chickpea Transcriptome Database (CTDB)	A catalog of transcription factor families and their expression profiles are available in the database and a resource for the discovery of functional molecular markers	http://www.nipgr.ac.in/ctdb.html
	CicArVarDB	A repository of 1.9 million variations (SNPs and InDels) anchored on eight pseudomolecules in a custom database	http://cicarvardb.icrisat.org/
	CicArMiSatDB	Database provides detailed information on SSRs along with their features in the genome	http://cicarmisatdb.icrisat.org/

Pigeonpea	PpTFDB	A pigeonpea transcription factor database for exploring functional genomics in legumes	http://14.139.229.199/PpTFDB/Home.aspx
	Pipemicrodb	Microsatellite database and primer generation tool for pigeonpea genome	http://webapp.cabgrid.res.in/pigeonpea/

The initial maize reference genome sequence (2.3-billion-base; B73 RefGen_v1) was developed from the public sector inbred B73, a widely used female parent for maize hybrids and genetics. The first draft of the B73 genome assembly was annotated with two different sets of genes, a high confidence filtered gene set containing roughly 32,000 putative gene models and a lower confidence working gene set which contained 110,000 putative gene models spread across 10 chromosomes (Schnable et al., 2009). While this initial draft was assembled using a BAC tiling path and Sanger sequencing of individual BACs, more recent updates of the genome assembly for B73 have employed *de novo* assembly from long single molecule sequencing technologies (B73 RefGen_v4; Jiao et al., 2017). Subsequent to the sequencing of B73, additional assemblies were generated for several maize inbreds including Mo17 (Yang et al., 2017b; Sun et al., 2018), W22 (Springer et al., 2018), HZS (Li et al., 2019b), SK (Yang et al., 2019), K0326Y (Li et al., 2020b), as well as for an accession of *Z. mays* ssp. *mexicana*, a close wild relative of domesticated maize (Yang et al., 2017b).

Whole genome resequencing and genotyping-by-sequencing (GBS) of a large number of maize inbreds has contributed to the development of several versions of maize haplotype maps (Gore et al., 2009; Chia et al., 2012; Bukowski et al., 2018. However, comparative analyses across different maize genomes have been limited by the challenge of determining differences in structure, sequence content, or gene content between the genome assemblies of different inbreds (Anderson et al., 2019). Sequence analysis of 75 wild, landrace and improved maize lines provided evidence of recovery of diversity after domestication, likely introgression from wild relatives, and evidence for stronger selection during domestication than improvement (Hufford et al., 2012).

Recent studies have begun to sequence and annotate groups of inbreds together using common sequencing strategies and software tools for assembly and annotation. A group of four European flint lines (EP1, F7, DK105 and PE0075) were sequenced and assembled as part of a single project (Haberer et al., 2020). The genomes of the 25 founder lines of the maize nested association population, selected to capture the maximum amount of the total diversity present in maize which could be grown in temperate environments (Yu et al., 2008), were sequenced, assembled, and annotated using a common software pipeline with the assemblies released to the community in early 2020 (NAM Genomes Project, 2020). Sequencing, assembling, and annotating genomes using common software programs controls for many of the biases present in comparisons across genome sequence assemblies for different individuals generated using different approaches. However, questions on optimal computational approaches to both represent the maize pan-genome defined by these many genome assemblies, as well as how best to employ these combined assemblies to support gene-to-phenotype associations and crop improvement, remain.

Approaches to pan-genome representation and application are also being developed in animal systems (Sherman and Salzberg, 2020), however the high degree of sequence divergence and structural divergence present in intergenic space between different maize haplotypes (Fu and Dooner, 2002; Wang and Dooner, 2006) makes most approaches from mammalian systems computationally intractable to apply to maize and other crop species with large genomes. One approach being explored in maize which does appear to be computationally tractable and provide substantial benefits above single reference based genomic analyses is the use of Practical Haplotype Graphs (Franco et al., 2020). There are several public databases (Table 1) such as MaizeDIG (hosts information on 10 different maize reference genomes), MaizeGDB (hosts genome metadata, RNA-seq, proteomics, synteny, and large-scale diversity), and MODEM (designed to promote a better understanding of maize genetic architecture and deep functional annotation of the complex maize genome) enables the use of comprehensive information for maize genetics research and breeding applications.

Bread wheat is an allohexaploid species (2*n* = 6*x* = 42, AABBDD genomes), formed from the combination of three interrelated diploid genomes. Hybridization and homoploid speciation of *T. urartu* (donors of the A genome) and *Ae. speltoides* (donor of B genome) generated *Ae. tauschii* (donor of the D genome). Hybridization between *T. turgidum* and *Ae. Tauschii* gave rise to the hexaploid *T. aestivum* (see Venske et al., 2016). The allohexaploid nature and large genome size (17Gb ∼40 times the genome of rice) made it challenging to decode the genome. However, the International Wheat Genome Sequencing Consortium (IWGSC) developed a high-quality reference genome sequence of the bread wheat cultivar Chinese Spring (CS) 16 years after the initial drafts of the rice genome (International Wheat Genome Sequencing Consortium (IWGSC, 2018). The wheat genome, with 124,201 protein coding genes across the 21 chromosomes, can serve as a model for understanding the mechanisms of polyploidy evolution, domestication, genetic and epigenetic regulation of homoeolog expression, as well as defining its genetic diversity and breeding on the genome level. Multiple versions of genome sequences of CS were reported by several groups with their special strategies (see Guan et al., 2020). The draft genomes of *Ae. tauschii* (DD genome, 50,264 protein coding genes) and *T. urartu* (AA genome; 53,056 protein coding genes) were reported in 2013 (Jia et al., 2013; Ling et al., 2013). Two reference quality assemblies of *Ae. tauschii*, 1 and 2, were published in 2017 (Luo et al., 2017; Zhao et al., 2017), while the reference quality assembly of *T. urartu* was reported in 2018 (Ling et al., 2018). The two tetraploid wheat wild emmer wheat (Avni et al., 2017) and durum wheat (Maccaferri et al., 2019; 91,097 protein coding genes) were sequenced in 2017 and 2019, respectively.

Based on analysis of whole genome sequencing of 93 accessions of bread wheat (including its diploid and tetraploid progenitors) and 90 published exome-capture data, Cheng et al. (2019) reported that the B sub-genome has more variations than the A and D sub-genomes, including SNPs and deletions. Targeted sequencing of 890 diverse accessions of hexaploid and tetraploid wheat indicated that historic gene flow from wild relatives made a substantial contribution to the adaptive diversity of modern bread wheat through increased genome wide diversity including the regions harboring major agronomic genes (He et al., 2019). The genomic regions or QTLs associated with footprints of modern wheat breeding were reported by studying 79,191 accessions from the CIMMYT and ICARDA germplasm banks that originated from 109 countries (Sansaloni et al., 2020). Several genomic databases like GrainGenes and other databases related to Triticeae or cereal species like CerealsDB (Wilkinson et al., 2020) host the vast genome and genetic information for wheat research and improvement (Table 1).

In the case of barley, a chromosome-scale genome assembly has been reported for the US spring six-row malting cultivar Morex using a hierarchical approach (Mascher et al., 2017). Furthermore, an improved high-quality genome assembly of the Tibetan hulless barley (3.89-Gb; 36,151 predicted protein-coding genes) showed high gene completeness and high collinearity of genome synteny with the previously reported barley genome and will also serve as a key resource for studying barley genomics and genetics (Zeng et al., 2015, 2018). In addition, Liu et al. (2020a) reported the genome of wild species of barley (AWCS276), which was comprised of 4.28 Gb genome and 36,395 high‐confidence protein‐coding genes. Comparative analysis of the AWCS276 genome with the Morex genome identified more genes involved in resistance and tolerance to biotic and abiotic stresses in the wild barley (Liu et al., 2020a).

Deep sequencing of several germplasm lines earlier provided insights into environmental adaptation of geographically diverse barley landraces and wild relatives (Russell et al., 2016), the origin and evaluation of qingke barley in Tibet (Zeng et al., 2018) and the genetic basis of adaptation in barley (Bustos‐Korts et al., 2019). Furthermore, genome sequences of five 6000-year-old barley grains excavated at a cave in the Judean Desert close to the Dead Sea were reported by Mascher et al. (2016). Based on comparison to whole-exome sequence data from a diversity panel of present-day barley accessions, this study showed the close affinity of ancient samples to extant landraces from the Southern Levant and Egypt, consistent with a proposed origin of domesticated barley in the Upper Jordan Valley. Illumina enrichment sequencing of 344 wild and 89 domesticated lines representing global barley diversity, provided 137 signatures of selective sweeps regions that contained candidate domestication genes responsible for different biological processes, such as light signaling regulation, circadian clock and carbohydrate metabolism pathways (Pankin et al., 2018). GBS of 22,626 barley collection at Leibniz Institute of Plant Genetics and Crop Plant Research (IPK), besides providing comprehensive insights into the global diversity of the domesticated barley, also enabled identification of candidate duplicates and highlighted collection gaps (Milner et al., 2019). Further, in combination with phenotypic data for many accessions, GBS data are a permanent resource for investigating the genes underlying crop evolution and selection for agronomic traits (https://bridge.ipk-gatersleben.de/#start; König et al., 2020). Databases like barleyGenes (access to the predicted genes from an assembly of whole-genome-shotgun sequence from barley), BARLEX (central repository and hub of genomic sequences of barley sequencing efforts), bex-db (a Bioinformatics workbench for comprehensive analysis of barley-expressed genes) and BRIDGE (visualizing Barley Genebank Genomics) are available to the barley research community in addition to GrainGenes and other Triticeae/ cereal species (Table 1).

For sorghum, a reference genome based on the elite grain inbred BTx623 was generated using whole genome shotgun sequencing in 2009 (Paterson et al., 2009) and further enhanced in 2018 to improve assembly quality and incorporate a further 29.6Mb of sequence (McCormick et al., 2018), in total identifying 34,211 protein-encoding genes. Strong racial structure and a complex domestication history involving two distinct domestication events were uncovered by whole genome re-sequencing of 44 sorghum germplasm lines representing the major races of *S. bicolor* (Mace et al., 2013). Recently, two additional high-quality reference genomes have been developed; one based on the archetypal sweet line, ‘Rio’, with 35,467 protein-encoding genes identified (Cooper et al., 2019); and one based on the elite line RTx430, with 34,211 protein-encoding gene models identified (Deschamps et al., 2018). Comprehensive analysis of 44 sorghum genotypes (including 18 landraces and seven wild and weedy sorghums along with two *G. margaritiferums* and two progenitors *S. propinquum* genotypes provided 128 genes displaying signatures of purifying selection, gene targets to improve nitrogen use efficiency in sorghum (Massel et al., 2016). Sequencing of six *S. bicolor* accessions from southwest China revealed that these accessions contained a large number of high‐confidence genes, with Hongyingzi in particular possessing 104 unique genes (Yan et al., 2018). SorghumFDB (a dynamic network of multidimensional biological relationships, comprised of the co-expression data, protein–protein interactions and miRNA-target pairs), SorGSD (a web-based query interface to search or browse SNPs from individual accessions, or to compare SNPs among several lines), SbGDB (helps in annotating the *Sorghum bicolor* genome using yrGATE gene structure annotation tool) are some key databases available in case of sorghum (Table 1).

The draft genome of pearl millet, a staple food for more than 90 million farmers in arid and semi-arid regions of sub-Saharan Africa, India and South Asia, based on the genotype Tift 23D_2_B_1_-P1-P5, was assembled by Varshney et al. (2017a) reporting 38,579 protein coding genes. In the same study, sequencing of 994 pearl millet lines that include 345 pearl millet inbred germplasm association panel (PMiGAP) lines, 31 wild accessions representing seven countries (Mali, Mauritania, Senegal, Sudan, Chad, Mali and Niger) and 580 hybrid parental [maintainer (B-) and restorer (R-) lines was also reported. This study provided useful insights into domestication and crop plasticity including the role of wax biosynthesis genes in tolerance to heat and drought. Resequencing data enabled to establish 1054 marker trait associations for 15 agronomic traits. In addition, subsets of the genome-wide SNPs were used for genomic prediction, and for defining heterotic pools and predicting hybrid performance (Philipp et al., 2016).

### 2.2 Legumes

In the case of soybean, several studies were conducted using *de novo* genome assembly and re-sequencing approaches in cultivated and wild soybean accessions. A preliminary draft of the reference genome of Williams 82 (Wm82), the cultivated soybean, was first provided by Schmutz et al. (2010). Kim et al. (2010) reported a draft genome sequence of undomesticated ancestor of *G. max,* the *G. soja* (accession IT182932; 915.4 Mb), representing a coverage of 97.65 % of the *G. max* genome sequence. The genomic structure of Japanese soybean was similarly characterized by sequencing a leading Japanese cultivar Enrei (Shimomura et al., 2015). Based on a more recent genome assembly of wild soybean accession W05 (1013.2 Mb), Xie et al. (2019) identified an inversion at the locus determining seed coat color as well as a region containing copy number variations of the *Kunitz trypsin inhibitor* (*KTI*) genes. Recently, Valliyodan et al. (2019) developed genome assembles for three soybean accessions Wm82, Lee (PI 548656) and *G. soja* accession PI 483463. In addition, by investigating five domestication loci, they also identified two different alleles with functional differences between *G. soja* and the two domesticated accessions. Recently, *de novo* genome assemblies for 26 representative soybeans (selected from 2898 deeply sequenced accessions) were released. A pangenome was constructed using these assembled genomes together with three previously reported genomes, which successfully identified numerous genetic variations that cannot be detected by direct mapping of short sequence reads onto a single reference genome (Liu et al., 2020b).

These studies together provided a foundation for subsequent exploration of soybean genomes to understand domestication and also provide tools for further crop improvement. For instance, Lam et al. (2010) conducted a genomic comparison of *G. max* and *G. soja* populations using whole-genome re-sequencing to reveal high diversity among wild genomes. In a subsequent study, Chung et al. (2014) reported sequencing and analysis of ten *G. max* and six *G. soja* accessions from Korea. Furthermore, re-sequencing of 302 soybean accessions including 62 wild soybeans (*G. soja*), 130 landraces and 110 improved cultivars provided insights into genes involved in soybean domestication (Zhou et al., 2015). Similarly, re-sequencing of 809 soybean accessions and characterization of 84 agronomic traits (Fang et al., 2017) reported 245 significant genetic loci for important agronomic traits and 14 oil synthesis-related genes responsible for fatty acid accumulation.

In the case of groundnut, the genome sequence of two diploid diploid progenitor species, *A. duranensis* V14167 (A genome) and *A. ipaensis* K30076 (B genome) was reported by the International Peanut Genome Initiative (IPGI) through the Peanut Genome Consortium (PGC) (Bertioli et al., 2016). In addition, Chen et al. (2016) decoded the genome of *A. duranensis* PI475845 (A genome) and Lu et al. (2018) reported the genome of *A. ipaensis* ICG 8206 (B genome). In 2018, the genome assembly was made available for the allotetraploid wild groundnut (∼2.62 Gb; 20 pseudomolecules), *A. monticola* PI 263,393, which is considered either the direct progenitor for the cultivated tetraploid groundnut or as an independent derivative between the cultivated groundnut and wild species (Yin et al., 2018). Finally, for cultivated and tetraploid groundnut, two reference genomes for subsp. *fastigiata* (Chen et al., 2019; Zhuang et al., 2019) and one for subsp. *hypogaea* (Bertioli et al., 2019) were reported.

For common bean, a reference genome 473 Mb of the 587-Mb genome was assembled in 11 chromosome-scale pseudomolecules for an inbred landrace (G19833) derived from the Andean pool (Schmutz et al., 2014). Two independent domestication events of common bean were confirmed based on sequencing of 60 wild individuals and 100 landraces from the genetically differentiated Mesoamerican and Andean gene pools. Less than 10 % of the 74 Mb of sequence putatively involved in domestication was shared by the two domestication events (Schmutz et al., 2014). The genome of Mesoamerican common bean BAT93 encompassing 549.6 Mb with 81 % of the assembly was anchored to eleven linkage groups (Vlasova et al., 2016). Stable associations for seed size, flowering time and harvest maturity traits were reported based on sequencing and phenotyping of 683 landraces and breeding line collections (Wu et al., 2020). Whole-genome sequencing of 37 varieties belonging to *P. vulgaris*, *P. acutifolius* (A. Gray), and *P. coccineus* L. revealed a large number of inter–gene pool introgressions and enabled mapping of interspeciﬁc introgressions for disease resistance in breeding lines (Lobaton et al., 2018).

In the case of cowpea, a preliminary draft assembly and BAC sequence assemblies of IT97 K‐499‐35 were generated from short-read sequences (Muñoz‐Amatriaín et al., 2017), followed by an improved assembly by Lonardi et al. (2019) using single‐molecule real‐time sequencing, optical and genetic mapping, and an assembly reconciliation algorithm Nearly half of the 519 Mb assembled sequence of the 641 Mb genome (determined by flow cytometry) is composed of repetitive elements, and comparative analysis of these elements revealed that genome size differences between published *Vigna* genome sequences are mainly due to differences in the amount of Gypsy retrotransposons. Recently, the genome sequence of the Asparagus bean (*V. unguiculata* ssp. *sesquipedialis*) a warm-season and drought-tolerant subspecies of cowpea was reported to contain 42,609 protein-coding genes and 3579 non-protein-coding genes in 632.8 Mb genome. In addition, CowpeaPan (https://phytozome-next.jgi.doe.gov/cowpeapan/) provides an interface to access annotated genome assemblies of seven diverse cultivated cowpeas.

In the case of chickpea, a draft genome sequence was generated for the kabuli genotype, CDC Frontier, by Varshney et al. (2013a). In parallel, genome assembly was also reported for a desi genotype ICC 4958 (Jain et al., 2013). The CDC Frontier genome assembly was comprised of 544.73 Mb of genomic sequence in scaffolds representing 73.8 % of the total genome (738.09 Mb) and 28,269 protein-coding genes. Following the availability of both draft genomes, efforts were made to improve the genome assemblies. The desi genome assembly was improved by generating additional sequence data (Parween et al., 2015) and a chromosomal genomics approach (Ruperao et al., 2014). The improved genome assembly of ICC 4958 was comprised of 30,257 protein-coding genes and a 2.7-fold increase in length of pseudomolecules. The genome assembly of *C. reticulatum,* the wild progenitor of chickpea, was also reported with 78 % (327 Mb) of this assembly was assigned to eight linkage groups and comprising of 25,680 protein-coding genes covering more than 90 % of the predicted gene space (Gupta et al., 2017). In addition, several germplasm sets have been targeted for sequencing and analysis. For instance, sequencing of 35 parental lines of different mapping populations in chickpea provided several genome-wide variations like SNPs, indel and structural variations that can be used for trait dissection (Thudi et al., 2016a). Sequencing of 129 released varieties of chickpea in 14 countries provided insights into the enhanced genetic diversity in both desi and kabuli varieties released after 2002 (Thudi et al., 2016b). Further, apart from providing insights into migration history of chickpea, sequencing of 429 lines also provided 262 marker-trait association for 13 traits related to tolerance to drought and heat tolerance (Varshney et al., 2019). Genome wide association studies based on sequencing of 132 chickpea varieties and advanced breeding lines phenotyped for 13 traits related to tolerance to drought tolerance in western Australia provided several SNPs from auxin-related genes, including auxin efflux carrier protein (PIN3), p-glycoprotein, and nodulin MtN21/EamA-like transporter, were significantly associated with yield and yield-related traits under drought-prone environments (Li et al., 2018).

For pigeonpea, an initial draft genome assembly was developed for Asha variety (ICPL 87,119) (Varshney et al., 2012). In this assembly, 72.7 % (605.78 Mb) of the 833.07 Mb genome was assembled into scaffolds and 48,680 protein coding genes were predicted. In parallel, Singh et al. (2012) also reported the draft genome of Asha variety using long sequence reads of 454 GS-FLX sequencing. Employing assembly reconciliation approaches, Marla et al. (2020) reported a finished assembly with reduced number of gaps and improved genome coverage of 82.4 %. Sequencing of 20 pigeonpea accessions led to the development of a first-generation hapmap and unique molecular signatures that hold great relevance in terms of varietal identification and genotypes adapted to particular agro-ecologies (Kumar et al., 2016). Subsequently, based on sequencing of 292 *Cajanus* accessions, encompassing breeding lines, landraces and wild species, several genomic regions that were likely targets of domestication and breeding, and indicative of center of origin and migration routes (Varshney et al., 2017b). In addition, GWAS studies revealed associations between several candidate genes for flowering time control, seed development and pod dehiscence (Varshney et al., 2017b; Zhao et al., 2020a). Recently, a pangenome of 622.88 Mb with 55,512 protein-coding genes was constructed using sequencing data from the reference cultivar and 89 pigeonpea accessions (70 from South Asia, 8 from sub‐Saharan Africa, 7 from South‐East Asia, 2 from Mesoamerica and 1 from Europe) (Zhao et al., 2020a).

For several legume crops, generic databases such as the Legume Information System (LIS https://legumeinfo.org/; LegumeIP, https://plantgrn.noble.org/LegumeIP; and Know Pulse, https://knowpulse.usask.ca) have been developed for providing genomic information. In some cases, dedicated legume specific databases have also been developed, some of which are presented in Table 1.

## 3 Genotyping platforms for accelerating trait genetics and breeding applications

**Fig. 1:**
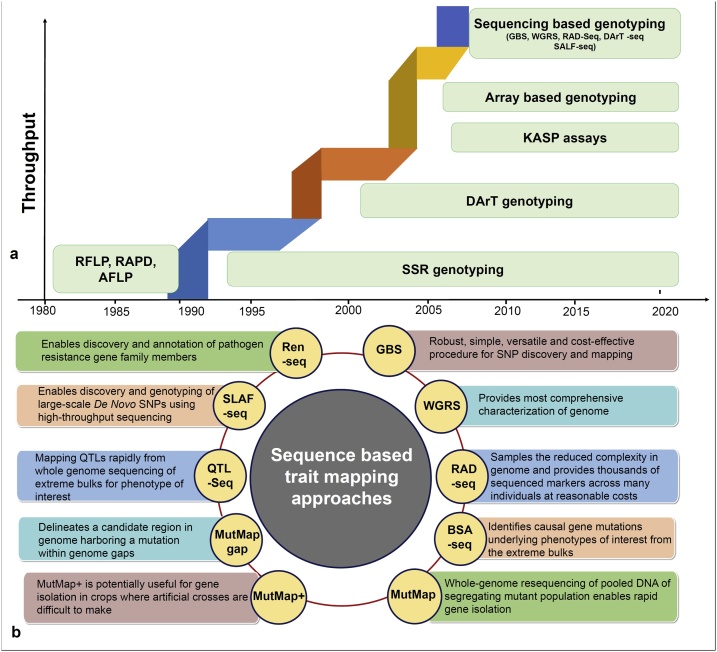
Genotyping platforms and key sequencing-based trait mapping approaches. (a) Molecular markers and genotyping platforms, during the last there decades, have evolved significantly. While throughput has been increasing and cost-per-marker datapoint has been decreasing over the years. (b) Availability of reference genome sequences, the cost-effective genotyping platforms, and a range of genetic populations have provided new faster sequencing-based trait mapping approaches [like geotyping by sequencing (GBS), whole genome resequencing (WGRS), restriction site associated DNA Seq (RAD-seq), bulked segregant analysis-sequencing (BSA-seq) MutMap, MutMap+, MutMap-Gap, QTL-seq, Specific locus amplified fragment sequencing (SLAF-seq), resistance gene enrichment sequencing (RenSeq)]. With these platforms and trait mapping approaches, it has been possible to map target traits for breeding programmes in time- and cost- effective manner in recent years.

During the last three decades, due to advances in genetics, genomics, automation, robotics and bioinformatics, a range of marker genotyping platforms have become available (Fig. 1a). These marker types include restriction fragment length polymorphisms (RFLPs), randomly amplified polymorphic DNAs (RAPDs), amplified fragment length polymorphisms (AFLPs), SSRs, Diversity Array Technology (DArTs) and single nucleotide polymorphisms (SNPs). Among these marker types, SNPs are the most abundant and stable genetic variations in the genome and are amenable to high throughput detection and genotyping and are used extensively in plant breeding. The availability of millions of SNPs from genomes and germplasm sequencing projects has enabled availability of a plethora of cost effect approaches for high throughput SNP genotyping for genetic and genomic studies. A comparison of different types of SNP genotyping platforms have been provided in several earlier studies (Mir et al., 2013; Rasheed et al., 2017). Therefore, we provide here only the key genotyping platforms that have been developed and are available in targeted cereal and legume crops (Table 2). As an alternative genotyping platform for medium- and high-density arrays, 20 K and 40 K SNP arrays were developed in maize through genotyping by target sequencing (GBTS) and in-solution capture, which are cost-effective, array-flexible and user-friendly (Guo et al., 2019; Xu et al., 2020). The same set of designed arrays can be used to capture different sets of markers by sequencing at different depths.

**Table 2:** List of SNP arrays available in select cereals and legumes.

Crop	Size	Technology	Reference
Rice	7 K	Illumina Infinium	Morales et al., 2020
	50 K	Affymetrix Axiom	Bi et al., 2019
	6 K	Illumina Infinium	Thomson et al., 2017
	700 K	Affymetrix Custom GeneChip	McCouch et al., 2016
	50 K	Affymetrix Axiom	Singh et al., 2015
	50 K	Illumina Infinium BeadChip	Chen et al., 2014
	6 K	Illumina Infinium BeadChip	Yu et al., 2013
	44 K	Affymetrix SNP 6.0	Zhao et al., 2011
	44 K	Affymetrix Custom	Tung et al., 2010

Maize	20 K	GBTS (Genotyping by Target Sequencing)	Guo et al., 2019
	55 K	Affymetrix Axiom	Xu et al., 2017
	600 K	Affymetrix Axiom	Unterseer et al., 2014
	50 K	Illumina Infinium	Ganal et al., 2011

Wheat	35K	Affymetrix Axiom	King et al., 2017
	7 K	DArTseq	Vikram et al., 2016
	820 K	Affymetrix Axiom	Winfield et al., 2016
	90 K	Illumina Infinium	Wang et al., 2014
	9 K	Illumina Infinium	Cavanagh et al., 2013

Barley	50 K	Illumina Infinium	Bayer et al., 2017
	3 K	Illumina Infinium	Philipp et al., 2016
	9 K	Illumina Infinium	Comadran et al., 2012

Soybean	6 K	Illumina Infinium	Song et al., 2020
	180 K	Affymetrix Axiom	Jeong et al., 2019; Lee et al., 2015b
	8 K	Illumina Infinium BeadChip	Wang et al., 2018a,b
	355 K	Affymetrix Axiom	Wang et al., 2016
	50 K	Illumina Infinium BeadChip	Song et al., 2013

Peanut	58 K	Affymetrix Axiom	Pandey et al., 2017

Cowpea	51K	Illumina Infinium	Muñoz‐Amatriaín et al., 2017

Common bean	768	Illumina Goldengate assay	Blair et al., 2018
	6 K	Illumina Infinium BeadChip	Song et al., 2015

Chickpea	50 K	Affymetrix Axiom	Roorkiwal et al., 2018

Pigeonpea	50 K	Affymetrix Axiom	Saxena et al., 2018

Array-based genotyping and sequencing- based genotyping approaches e.g. whole genome re-sequencing (WGRS), genotyping-by-sequencing (GBS), restriction site associated DNA sequencing (RAD-Seq) (Fig. 1a) have been extensively used for genetic diversity analyses, identification of QTLs via bi-parental mapping, genome-wide association studies, marker-assisted selection, and genomic prediction studies. However, when only a few SNP markers need to be used, for example in forward breeding applications (Varshney, 2016), array-based genotyping platforms, generally ranging from $25 to $100 per DNA sample, are not cost-effective. To address these issues, the High-ThroughPut Genotyping (HTPG) project, supported by Bill and Melinda Gates Foundation (BMGF) and implemented by ICRISAT (http://cegsb.icrisat.org/high-throughput-genotyping-project-htpg/) in collaboration with various other CG centers and Intertek-Agritech have provided a platform for genotyping upto 10 SNPs (diagnostic markers) for forward breeding applications. By using this platform, breeding programs can access genotyping of breeding populations at the rate of US$ 1.5–2.0 per sample, including DNA extraction costs (Bohar et al., 2020).

## 4 Novel trait mapping approaches

Molecular markers associated with traits are pre-requisite for undertaking marker-assisted selection and marker-assisted backcrossing programs. In the pre-genome sequencing era, marker-trait association studies were primarily conducted using bi-parental and association mapping populations. However, these studies were time and cost intensive, orders of magnitude higher than contemporary options, as they needed to genotype each population with SSR or SNP markers. The availability of reference genomes have made it possible to undertake trait mapping studies at a faster pace and much reduced costs. Several studies in recent past have proposed trait mapping by sequencing or genotyping of either extreme pools or individuals segregating for qualitative and quatitative phenotypes from bi-parental populations. These approaches include SHOREmap, X-QTL, Next Generation Mapping, BSR-seq, MutMap, MutMap+, MutMap-Gap, QTL-seq and SLAF-seq (Schneeberger et al., 2009; Ehrenreich et al., 2010; Austin et al., 2011; Schneeberger and Weigel, 2011; Liu et al., 2012; Abe et al., 2012; Takagi et al., 2013; Fekih et al., 2013; Zou et al., 2016). In principle MutMap (pooling based on a segregating trait) and QTL-seq (selecting phenotypic extremes) are essentially the same as the ‘classical’ bulk segregant analyses (BSA), except that markers are generated by high-throughput sequencing of pooled DNA, and a large number of SNPs are mapped onto the genome, and any populations can be used to create bulked samples (Zou et al., 2016) (Fig. 1b). In both cases, the mapping resolution depends on the number of individuals in the bulk and if there are not enough number of samples in the pools, it is difficult to identify the causal SNPs associated with the trait. These approaches have been used for mapping a number of traits in several cereal and legume crops. In the following sections, a few studies using MutMap and QTL-Seq approaches for trait mapping in crop species are provided.

The MutMap approach enabled the identification of loss-of-function mutants in ethyl methanesulfonate (EMS) mutant lines of a local elite cultivar, ‘Hitomebore’. Furthermore, a mutation responsible for salt tolerance, named Hitomebore salt tolerant 1 (*hst1*) gene in rice (Takagi et al., 2015). In another study, the *CAO1*gene associated with pale green color leaf was mapped using MutMap in rice (Abe et al., 2012). Similarly, in maize, mapping-by-sequencing, similar to MutMap, enabled the identification of a mutation in *ZmCLE7* underlying fasciation in an EMS mutant population (Tran et al., 2020). MutMap approach was also used to map GDSL like lipase/acylhydrolase associated with drought tolerance in sorghum (Jiao et al., 2018). In soybean, the *lm1* locus governing mutant phenotype of *spl-1* was identified in 3.15 Mb genomic region on chromosome 04 through MutMap analysis, that was further verified and fine mapped by SSR marker-based genetic mapping (Al Amin et al., 2019). MutMap+, a variant of MutMap approach, is suitable for identifying mutations that cause early development lethality, sterility, or generally hamper crossing and this does not involve artificial crossing between mutants and the wild-type parental line (Fekih et al., 2013). Using MutMap+ approach, novel mutant alleles for fine-tuning of cooked rice texture were identified in a rice starch branching enzyme IIb gene (Nakata et al., 2018). Similarly, in maize, mapping-by-sequencing, an approach similar to MutMap, enabled identification of a mutation in *ZmCLE7* underlying fasciation in a EMS mutant population (Tran et al., 2020). MutMap was used also to map GDSL like lipase/acylhydrolase associated with drought tolerance in sorghum (Jiao et al., 2018).

QTL-seq has been extensively used for rapid identification of causal SNPs and genes in several crops. In rice, QTL-seq identified candidate genes regulating grain weight, grain length, *Pi65(t),* a novel broad spectrum resistance gene to rice blast, novel QTLs qDTH4.5 and qDTH6.3 conferring late heading under short-day conditions (Takagi et al., 2013; Daware et al., 2016; Ogiso-Tanaka et al., 2017; Yaobin et al., 2018). Furthermore, QTL-seq coupled with RNA-seq at the bud burst stage in rice enabled identification of a major QTL and candidate gene for salt tolerance (Lei et al., 2020). QTL-seq identified cooked grain elongation QTLs near soluble starch synthase and starch branching enzymes in rice (Arikit et al., 2019). Owing to the large genome size of barley, Exome QTL-seq, a combination of exome sequencing and QTL-seq was used to map black lemma and pericarp (*Blp*) loci and QTLs for resistance to net blotch disease (caused by the fungus *Pyrenophora teres)* using doubled haploid barley lines (Hisano et al., 2017). In sorghum, using a combination of BSA and deep sequencing technologies, researchers were used to fine map stem water controlling locus, *qSW6* to 339 kb region containing 38 putative genes (Han et al., 2015).

In the case of legumes, QTL‐seq approach has been successfully deployed in case of soybean, groundnut, chickpea and pigeonpea. For instance, QTL-seq combined with linkage mapping was used for fine mapping a wild soybean allele characteristic of greater plant height (Zhang et al., 2018a). Similarly, two qualitative genes, *D1* and *D2*, controlling cotyledon color of seed in soybean were mapped using BSA-seq/QTL-seq (Song et al., 2017). In chickpea, two genes (*Ca_04364* and *Ca_04607*) for 100 seed weight and one gene (*Ca_04586*) for total dry root weight to total plant dry weight ratio were identified using the QTL-seq approach (Singh et al., 2016). Similarly, one major genomic region harbouring a robust 100-seed weight QTL was identified using an intra-specific 221 chickpea mapping population (ICC 7184 × ICC 15,061). In another study, using a multiple QTL-seq strategy in chickpea identified regulatory and coding (non-synonymous/synonymous) novel SNP allelic variants from two *efl1* (early flowering 1) and *GI* (*GIGANTEA*) genes that regulate flowering time (Srivastava et al. (2017). In case of groundnut, the QTL-seq approach delineated the genomic regions and provided the candidate genes controlling shelling percentage (Luo et al., 2019), fresh seed dormancy (Kumar et al., 2020), purple testa color (Zhao et al., 2020b) rust and leaf spot resistance (Pandey et al., 2017). Similarly, in pigeonpea, QTL-seq (mentioned as Seq-BSA) was used to identify four candidate SNPs in four genes with fusarium wilt resistance and four candidate SNPs in three genes with sterility mosaic disease resistance.

Besides MutMap and QTL-seq, many other NGS-based approaches have been used with different names for trait mapping. For instance, a combination of BSA and SLAF-seq (Specific locus amplified fragment sequencing) enabled the identification of blast resistance gene(s) in Huazhan (HZ), a rice restorer line widely used in hybrid rice in recent years (Chen et al., 2016). Similarly, SLAF-seq approach identified a total of 27 QTLs for 100 seed weight, seed length, seed width and length to width ratio in groundnut (Zhang et al., 2019). Singh et al. (2016) proposed Indel‐seq approach, which is a combination of whole‐genome resequencing (WGRS) and bulked segregant analysis (BSA) and relies on the Indel frequencies in extreme bulks. This approach identified 16 Indels affecting 26 putative candidate genes for resistance to fusarium wilt and sterility mosaic disease in pigeonpea. We believe that the above mentioned approaches and several other new approaches based on NGS technologies will be in routine use for faster trait mapping that will accelerate molecular breeding in various crop species.

## 5 Genomics-assisted breeding

The genomics revolution has provided several tools to breeders for tailoring climate smart crops. Genomics assisted breeding (GAB) has been successfully deployed for combating biotic and abiotic stress in both cereals and legumes (Kole et al., 2015) and improvement of nutritional quality traits in agricultural crops (see Chandra et al., 2020). While deployment of markers and genomics technologies is in routine use in private sector and developed countries, several success stories of GAB have become available in public sector breeding programmes in developing countries. In the following sections, some examples of development of superior varieties/lines are presented.

### 5.1 Biotic stress resistance

To combat the emergence of virulent biotypes/strains of different plant pathogens research efforts needs to be focused on: (i) the development of new crop varieties with enhanced host resistance mechanisms; (ii) reconstituting the broken resistance over a period of time through pyramiding of multiple disease-resistant genes; and (iii) developing durable disease-resistant cultivars to the prevailing and emerging pathogen biotypes. With these objectives, superior lines have been developed in rice for: (a) bacterial blight resistance- Improved Pusa Basmati I, Improved Samba Mahsuri, Pusa 6A, Pusa 6B, Improved Lat, Improved Tapaswin, Improved Mangeumboye (Kottapalli et al., 2010; Singh et al., 2011; Dokku et al., 2013; Suh et al., 2013), (b) brown plant hopper resistance (e.g., Suh et al., 2011), and (c) blast resistance (e.g., Sanchez et al., 2000; Singh et al., 2001). Similarly, in the case of maize, MABC lines have been developed with enhanced resistance to southwestern corn borer (Willcox et al., 2002), European corn borer (Flint-Garcia et al., 2003), head smut (Zhao et al., 2012) and sorghum downy mildew (Sumathi et al., 2020). In wheat, during last two decades, more than 50 genes have been suggested for MAS, for diseases including powdery mildew, leaf rust, stem rust, stripe rust, cereal cyst nematode, hessian fly, wheat streak mosaic virus etc (Gupta et al., 2010; Dreisigacker et al., 2016). As a result, several molecular breeding lines have been developed with enhanced resistance to cereal cyst nematode resistance (Barloy et al., 2007), Fusarium head blight (Miedaner et al., 2009; Salameh et al., 2011), leaf rust (Nocente et al., 2007; Kumar et al., 2010; Yadawad et al., 2017; Randhawa et al., 2019), stem rust (Niu et al., 2011; Klindworth et al., 2012; Yadav et al., 2015), spot blotch (Vasistha et al., 2016), and others. Barley yellow mosaic virus disease resistance through molecular breeding has been a target in barley in Europe (Werner et al., 2005). In sorghum, success stories have become available for shoot fly resistance (Gorthy et al., 2017; Abinaya et al., 2019). Of particular note, four *Striga*-resistant varieties T1BC3 S4, AG6BC3 S4, AG2BC3 S4 and W2BC3 S4 were developed and released in Sudan (Mohamed et al., 2014). Furthermore, at the Institute for Agricultural Research (IAR), Samaru, Nigeria, using N13 as donor Striga resistance QTLs from N13 were introgressed into 10 farmer preferred varieties (SAMSORG17, SAMSORG40, SAMSORG43, SAMSORG14, SAMSORG39, SAMSORG41, DANYANA, CRS-01 and CRS-02). A total of 42 and 43 BC_2_F_1_ lines were developed in the background of DANYANA and SAMSORG39 which can be further evaluated for possible release as improved lines (Afolayan et al., 2019). Marker-assisted selection was used in pearl millet to develop improved lines with resistance to downy mildew (Sharma, 2001; Hash et al., 2006; Taunk et al., 2018) and drought tolerance (Yadav et al., 2005; Sehgal et al., 2015). Furthermore, six putatively improved HHB 197 hybrids were successfully tested in first year trials and two selected versions with higher yield and zero downy mildew incidence will be further tested in multi-location trials in India (Taunk et al., 2018).

Like cereals, molecular breeding efforts have delivered several improved lines for biotic stress resistance in legumes. In soybean, for soybean cyst nematode (SCN, *Heterodera glycines* Ichinohe) resistance, two sources of SCN resistance have been widely used, from the accessions PI 88788 (*rhg1-b*) and Peking (*rhg1-a* and *Rhg4*). By introgressing these resistance genes, varieties with enhanced SCN resistance have been developed in soybean (Santana et al., 2014). Ramalingam et al. (2020) developed soybean ILs with enhanced resistance to *Phytophthora* rot and powdery mildew diseases by introgressing *Rps2* and *Rmd-c* genes respectively. Broad-spectrum resistance against the existing strains of soybean mosaic virus (SMV) in China was achieved through pyramiding of three SMV resistance genes, *R_SC4_, R_SC8_*, and *R_SC14Q_* (Wang et al., 2017a, b). In groundnut, molecular breeding lines were developed with enhanced resistance to nematode (Chu et al., 2011), rust and late leaf spot (LLS) (Varshney et al., 2014a; Yeri and Bhat, 2016; Kolekar et al., 2017). Six cowpea mosaic virus (CpMV) resistant backcross progenies with 84.09–93.18 % background genome recovery and phenotype similar to C-152 were developed through MABC (Dinesh et al., 2018). ‘Moussa Local’, a local farmer-preferred purified variety from Burkina Faso was improved for drought tolerance, Striga and root-knot nematode resistance using IT93 K-503-1 and IT97 K-499- 35 as donors and genes six best backcross families from the two donors (Batieno et al., 2016). In addition, Striga resistant lines are also being developed from Melakh and IT97 K-499-39 (https://www.canr.msu.edu/legumelab/uploads/files/Diangar_Marker-assisted_Backcrossing.pdf). In common bean, two resistance genes, Co-5 and Co-42 for Anthracnose were effectively transferred to the BC_1_ population (Garzón et al., 2008). Subsequent studies have also selected for bruchid and virus resistance (Blair et al., 2010). In the case of chickpea, molecular breeding lines have been developed with enhanced resistance to Fusarium wilt and Ascochyta blight (Varshney et al., 2014b). Furthermore, Super Annigeri-1, an improved version of Annigeri- 1, and “Pusa Chickpea Manav” were developed by introgressing resistance for Fusarium wilt through MABC approach was successfully released in India (Mannur et al., 2019; https://icar.org.in/content/development-two-superior-chickpea-varieties-genomics-assisted-breeding; https://www.icrisat.org/genomics-assisted-breeding-delivers-high-yielding-wilt-resistant-chickpea-for-commercial-cultivation-in-central-india/). Efforts are underway in pigeonpea for introgressing resistance to Fusarium wilt and sterility mosaic disease (Saxena et al., 2020).

### 5.2 Abiotic stress tolerance

As abiotic stress tolerance is complex and generally governed by many QTLs unlike disease resistance, there are limited success stories in the development of superior lines for enhanced abiotic stress tolerance through molecular breeding efforts. In the case of rice, several improved lines have been developed for drought tolerance (Dixit et al., 2012a, b; Arai-Sanoh et al., 2014), submergence tolerance (SUB1) (Septiningsih et al., 2009, 2015) and salinity tolerance (Linh et al., 2012; Luu et al., 2012; Singh et al., 2018). Improved lines were developed for drought tolerance in maize (Ribaut, 2006), wheat (Todkar et al., 2020), barley (Baum et al., 2007), sorghum (Kassahun et al., 2010), and pearl millet (Kholová et al., 2016). Acid soils and aluminum (Al^3+^) toxicity hamper barley production, and the introgression of the HvAACT1 gene which confers Al resistance in barley resulted in the development of an Al-resistant line with 121 % more seeds than its isogenic line in soil-based assays using 12 % Al saturation (Soto-Cerda et al., 2013).

Drought tolerance has also been a target trait for enhancement in legumes. In this context, by using ICC 4958 as a donor, a “*QTL-hotspot*” containing QTLs for 12 traits was introgressed in the genetic background of JG11 (Varshney et al., 2013b). Based on multi-location trials of these MABC lines, the Geletu variety was recently released for commercial cultivation in Ethiopia (https://www.icrisat.org/first-ever-high-yielding-chickpea-variety-developed-using-marker-assisted-backcrossing-mabc-released-in-ethiopia/). Similarly using MABC in the genetic background of Pusa 372, a high yielding drought tolerant variety, Pusa Chickpea 10,216 was released in 2019 in India (https://icar.org.in/content/development-two-superior-chickpea-varieties-genomics-assisted-breeding; Bharadwaj et al., 2020). In addition, backcross progenies with enhanced drought tolerance were developed in different genetic backgrounds in India as well as Kenya and Ethiopia (see Thudi et al., 2014).

### 5.3 Quality and nutrition traits

In recent years, quality and nutrition traits have become priorities for several breeding programmes. For instance, molecular breeding lines have been developed for low amylose content (Tao et al., 2016), cooking and eating quality in rice (Ni et al., 2011). Several improved Quality Protein Maize cultivars like Vivek QPM-9, Pusa HM-4 Improved, Pusa HM-8 Improved, and Pusa HM-9, CML244Q, CMl246Q, CML349Q, CML354Q Improved, HQPM-1, HQPM-4, HQPM-5, and HQPM-7 were released for commercial cultivation in India (Gupta et al., 2013; Prasanna et al., 2020). In the case of wheat, MABC lines with enhanced grain protein content and pre-harvest sprouting tolerance were developed (Gupta et al., 2008; Vishwakarma et al., 2014, 2017). In the case of barley, where high quality malting barley varieties are preferred by brewers, Schmierer et al. (2004) developed one isogenic line (00–170) that has consistently produced high yield and high-malting quality profile.

Like cereals, similar efforts for improving quality or value addition have been undertaken in several legume crops. For instance, in the case of soybean, the presence of Kunitz trypsin inhibitor (KTI) in seeds necessitates pre-heat treatment of soy-flour, a step that not only enhances processing costs of the soy-based foods and feeds but also affects seed-protein quality and solubility. Six KTI free breeding lines of soybean were developed in the background of DS9712 and DS9814 using molecular marker-assisted backcross breeding approach (Kumar et al., 2015). Similarly, efforts to eliminate lipoxygenase-2, that causes off-flavour of soy products, from cultivar JS97-52 through marker assisted introgression of null allele of *Lox2* from PI596540 (*lox2lox2*) also provided significant improvement in seed longevity (Rawal et al., 2020). Earlier, PI086023 was used as the donor parent for lipoxygenase-2 and the first lipoxygenase-2 free soybean NRC109 was developed in India (Kumar et al., 2013). In another study, marker-assisted introgression of *cgy‐2*, a null phenotype version of the gene encoding the β‐conglycinin α‐subunit, from the donor line ‘RiB’ into the genetic background of the Chinese cultivar ‘Dongnong47’ (DN47), enabled development of allergen free seeds with enhanced nutritional value and food‐processing quality. In groundnut, by transferring of two *FAD2* mutant alleles from SunOleic 95R into the genetic background of ICGV 06110, ICGV 06142 and ICGV 06420, MABC lines with enhanced oleic acid ranging from 68 to 83 % were developed (Janila et al., 2016). As a result, the first set of high oleic acid varieties, Girnar 4 (ICGV 15083) and Girnar 5 (ICGV 15090) with about 80 % oleic acid were released for cultivation in six major groundnut growing states of India, namely Gujarat, Rajasthan, Karnataka, Tamil Nadu, Andhra Pradesh and Telangana (https://www.icar.org.in/content/icar-directorate-groundnut-research-develops-groundnut-variety-high-oleic-acid). Very recently, using GPBD4 as donor for foliar disease resistance and SunOleic 95R as donor for high oleic acid content, ILs were developed in the genetic background of three popular Indian cultivars (GJG 9, GG 20, and GJGHPS 1 (Shasidhar et al., 2020), which can be further used for pyramiding resistance to foliar diseases and high oleic acid content.

As mentioned earlier, we have presented here only a few success stories of molecular breeding as examples. We are aware that that there are many more such GAB studies that have reported development of lines for biotic stress resistance, abiotic stress tolerance and quality traits. Several reviews and books have also documented such studies. With the availability of genome sequences and faster trait mapping approaches we anticipate acceleration of GAB for a variety of applications across crop species.

## 6 Future perspectives

As mentioned in the above section, GAB has contributed to the development of improved climate resilient and high nutrition varieties in both cereals and legumes. A majority of these success stories have become available based on limited genomic resources, mainly molecular markers and genetic maps. In the post genome sequencing era, ample genomic resources such as genome sequence assemblies, germplasm sequencing data and gene expression atlases are available now. Specialized genetic populations such as multi-parent advanced generation intercross (MAGIC) and nested association mapping (NAM) populations have been generated in several cereal and legume crops. Similarly, high-throughput and cost- effective genotyping platforms and faster trait mapping approaches have become available. These efforts will further accelerate trait mapping, and with higher resolution than previously. Molecular markers/diagnostic markers associated with essential traits can be deployed in forward breeding applications using the HTPG platform in a cost-effective manner.

**Fig. 2:**
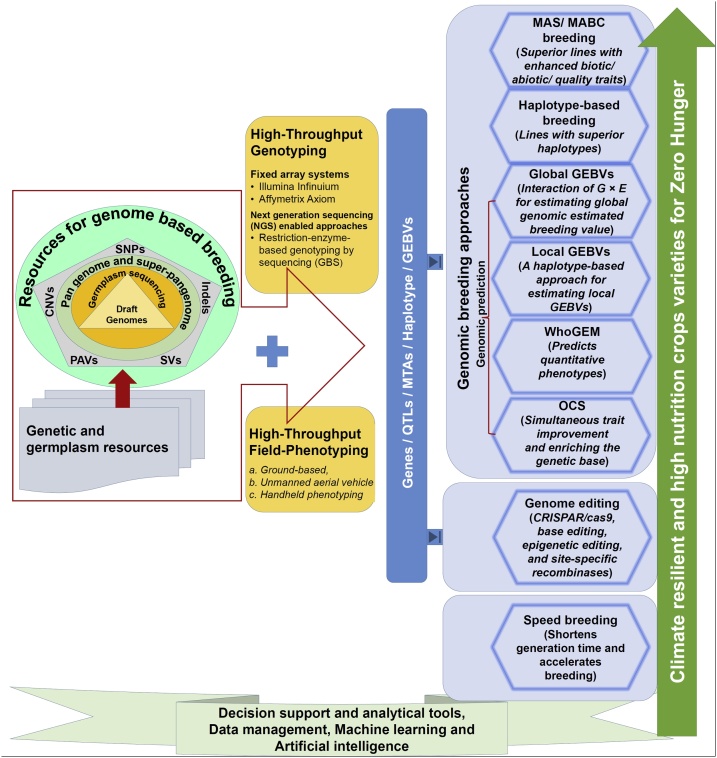
An integrated framework for using genomic resources for developing climate resilient and high nutrition crop varieties. During the last two decades ample genomic resources have been developed. The availability of draft genomes as well as sequence information from germplasm sets and specialized genetic populations bestowed the research community with millions of genome wide variations (SNPs, Indels, SVs, CNVs and PAVs) and pangenomes. Using high-throughput genotyping and high-throughput precise phenotyping approach, complex traits can be simplified at the genetic/ genome level by using sequencing-based trait mapping approaches, as mentioned in Fig. 1b. The genes, haplotypes, marker-trait association (MTA) and GEBVs can be used in genomic breeding or gene editing approaches. Genomic breeding approaches including MABC/ MAS, haplotype based breeding, and genomic prediction. We have shown four genomic prediction approaches namely global GEBVs, local GEBVs, WhoGEM and optimal contribution selection. The genomic breeding or gene editing approach can be combined with ‘speed breeding’ approach to reduce time in tailoring climate resilient and high nutrition crops.

In a recent review, deeper understanding and the deployment of 5 Gs (Genome assembly, Germplasm characterization, Gene(s)/ marker(s) associated with breeding trait, Genomic Breeding and Gene editing) was suggested for crop improvement (Varshney et al., 2020). While genome assemblies have become available in all target cereal and legume crops, germplasm characterization is underway in several crop species. Similarly, gene/marker identification efforts will be accelerated due to the availability of genomic and genetic resources and genotyping platforms. However, it is still of utmost important to have precise phenotyping for the germplasm being used for trait mapping. At present a range of trait phenotyping platforms are available (Jin et al., 2020). Comprehensive analyses of genotyping data and phenotyping data, depending on the population used, can provide genes/markers, haplotypes, genomic estimated breeding values that can be used in genomic breeding and gene editing approaches (Bohra et al., 2020). We believe that one or a combination of the following three genomic breeding approaches, namely MAS/MABC, haplotype-based breeding (HBB) and genomic prediction, can be used to develop superior lines. While MAS/MABC has been successfully used for product development, HBB has shown huge potential for trait improvement in rice (Abbai et al., 2019) and pigeonpea (Sinha et al., 2020). During recent years the accuracy and prediction of predicting phenotypes in genomics selection has been improved extensively through approaches including (i) estimating global GEBVs while considering interaction of marker and environment covariates (G × E) (Jarquín et al., 2014; Crossa et al., 2017), (ii) estimating haplotype/bin- based local GEBVs (Voss-Fels et al., 2019), (iii) WhoGEM approach that explores the relationships between phenotypes and admixture components, land types, admixture components × environment interactions, and controls for the environment (Gentzbittel et al., 2019), and (iv) optimal contribution selection method that enables simultaneous trait improvement and enriching the genetic base (Woolliams et al., 2015; Cowling et al., 2017). In case, a causal gene is available for a trait, gene editing approach can be used for the trait improvement (Zhang et al., 2018b). As in the breeder’s equation, the rate of genetic gain is inversely proportional to the breeding cycle time, recently proposed ‘speed breeding’ approach can reduce the breeding cycle (Watson et al., 2018; Ghosh et al., 2018). In speed breeding, rapid generation cycling through single seed descent method and prolonged light phase is adopted for breaking the vegetative phase. Therefore, speed breeding approach can be combined with any genomic breeding or gene editing approach to develop the improved lines at a faster pace. An integrated view of genomic resources together with different approaches for developing climate resilient and high nutrition crop varieties has been presented in Fig. 2.

Once an improved line is developed and released after undertaking multi-location trials in a country, it is essential to ensure delivering of the improved varieties to farmers’ hands. Therefore, appropriate seed delivery system should be developed for a successful varietal replacement rate (Atlin et al., 2017). It is also important to integrate better agronomy in farmers’ fields to realize the full genetic potential of improved genetics (Varshney et al., 2018). We very much hope that the knowledge generated and acquired during the last two decades and being generated now will bring a paradigm shift in breeding especially with emerging disciplines including machine learning and artificial intelligence. These efforts in an integrated and coordinated manner, will contribute to achieving Sustainable Development Goal 2 – Zero Hunger by accelerating genetic gains in crop improvement programs and delivering climate resilient and high nutrition crop varieties.

## CRediT authorship contribution statement

**Mahendar Thudi:** Writing - original draft, Writing - review & editing. **Ramesh Palakurthi:** Writing - original draft. **James C. Schnable:** Writing - review & editing. **Annapurna Chitikineni:** Writing - original draft. **Susanne Dreisigacker:** Writing - review & editing. **Emma Mace:** Writing - review & editing. **Rakesh K. Srivastava:** Writing - review & editing. **C. Tara Satyavathi:** Writing - review & editing. **Damaris Odeny:** Writing - review & editing. **Vijay K. Tiwari:** Writing - review & editing. **Hon-Ming Lam:** Writing - review & editing. **Yan Bin Hong:** Writing - review & editing. **Vikas K. Singh:** Writing - review & editing. **Guowei Li:** Writing - review & editing. **Yunbi Xu:** Writing - review & editing. **Xiaoping Chen:** Writing - review & editing. **Sanjay Kaila:** Writing - review & editing. **Henry Nguyen:** Writing - review & editing. **Sobhana Sivasankar:** Writing - review & editing. **Scott A. Jackson:** Writing - review & editing. **Timothy J. Close:** Writing - review & editing. **Wan Shubo:** Writing - review & editing. **Rajeev K. Varshney:** Conceptualization, Writing - original draft, Writing - review & editing.

## Declaration of Competing Interest

The authors declare they have no conflict of interest.
